# Comparison between molecular and histological *IDH*-wild-type glioblastoma and extensive subgroup analysis of *IDH*-wild-type astrocytic tumors without genomic glioblastoma-defining alterations

**DOI:** 10.1007/s11060-026-05637-w

**Published:** 2026-06-10

**Authors:** Anna M. Seifert, Sven Richter, André Sagerer, Ioana Lemnian, Sylvia Herold, Sascha Brückmann, Dimitrios Emmanouilidis, Majd Alkhatib, Ilker Y. Eyüpoglu, Erik A. Williams, Daniel P. Cahill, Tareq A. Juratli

**Affiliations:** 1 Department of Neurosurgery, Faculty of Medicine, University Hospital Carl Gustav Carus, TUD Dresden University of Technology, Fetscherstraße 74, 01307 Dresden, Germany; 2 Else Kröner Fresenius Center for Digital Health, Faculty of Medicine, TUD Dresden University of Technology, Dresden, Germany; 3 PathoNext GmbH, Molecular Pathology, Leipzig, Germany; 4 Department of Pathology, Faculty of Medicine, University Hospital Carl Gustav Carus, TUD Dresden University of Technology, Fetscherstraße 74, 01307 Dresden, Germany; 5 Foundation Medicine Inc, Cambridge, MA USA; 6 Department of Pathology and Laboratory Medicine, University of Miami, Sylvester Comprehensive Cancer Center, Miami, FL USA; 7 Laboratory of Translational Neuro-Oncology, Department of Neurosurgery, Massachusetts General Hospital, Harvard Medical School, Boston, MA USA; 8 Department of Neurosurgery, Massachusetts General Hospital, Harvard Medical School, Boston, MA USA; 9 National Center for Tumor Diseases (NCT), NCT/UCC Dresden, A Partnership Between DKFZ, Faculty of Medicine and University Hospital Carl Gustav Carus, TUD Dresden University of Technology, and Helmholtz-Zentrum Dresden-Rossendorf (HZDR), Dresden, Germany

**Keywords:** *IDH* mutation, Glioblastoma, *TERT* promoter, *Methylation profiling*, Molecular, Survival

## Abstract

**Purpose:**

This study compares clinical characteristics and survival between molecular (MolGBM) and histological *IDH*-wild-type (*IDH*-WT) glioblastoma (HistGBM), and further characterizes histological lower-grade *IDH*-WT astrocytic tumors without genomic GBM-defining alterations.

**Methods:**

Adult patients with histologically lower-grade *IDH-*WT astrocytoma (WHO grade 2–3) and available tumor tissue were included. Tumors were classified according to the 2021 WHO Classification of CNS tumors. Biopsy-only cases were excluded. *IDH1* and *TERT* promoter (*TERT*p) mutations were analyzed via Sanger and whole-exome sequencing (WES). *TERT*p-WT tumors underwent WES and subsequent DNA methylation profiling. Clinical, molecular, and outcome data were collected.

**Results:**

The cohort comprised 47 surgically resected histologically lower-grade *IDH*-WT astrocytic tumors. Thirty-nine fulfilled WHO 2021 criteria for MolGBM, mainly based on *TERT*p mutation (*n* = 36), while eight lacked GBM-defining molecular alterations. Compared with HistGBM (*n* = 54), MolGBM more frequently presented with seizures and showed a lower Ki-67 index. Median overall survival (OS) was 19.8 months in MolGBM and 14.6 months in HistGBM, without a significant difference in univariable analysis (*p* = 0.11). Patients aged ≥ 60 years showed longer overall survival in the MolGBM group (17.9 vs. 12.3 months; *p* = 0.0079). In multivariable Cox regression adjusted for age, extent of resection, and completion of the Stupp regimen, MolGBM was independently associated with more favorable OS (HR 0.40, 95% CI 0.24–0.67, *p* = 0.0005). The eight tumors lacking GBM-defining alterations showed longer survival and marked diagnostic heterogeneity.

**Conclusion:**

MolGBM showed comparable unadjusted OS but more favorable adjusted OS than HistGBM, supporting clinical and biological heterogeneity among molecularly defined *IDH*-WT diffuse glioma. *IDH*-WT, *TERT*p-WT lower-grade astrocytic tumors lacking GBM-defining alterations require comprehensive molecular characterization.

**Supplementary Information:**

The online version contains supplementary material available at 10.1007/s11060-026-05637-w.

## Introduction

Glioblastoma (GBM) is among the most common primary malignant brain tumors, accounting for 52.2% of all newly diagnosed malignant central nervous system (CNS) tumors. However, prognosis remains poor: median overall survival (OS) ranges from 9 to 15 months [1, 2].

Precise classification and molecular characterization are of critical importance, with genomic and epigenomic profiling forming the backbone of glioma diagnostics [3–6]. Historically, GBM was defined by the presence of necrosis or microvascular proliferation on histological examination. However, studies demonstrated that *IDH*-wild-type (-WT) astrocytic tumors harboring specific genomic alterations exhibit an aggressive clinical course comparable to that of classical GBM (HistGBM), even in the absence of histological high-grade features [7–9]. The 2021 update to the World Health Organization (WHO) classification of tumors of the CNS incorporates this fundamental paradigm shift: rather than relying solely on histological evaluation, genetic alterations with prognostic relevance now play a central role [10, 11]. Consequently, the cIMPACT-NOW working group has considered whether GBM can also be identified in histologically lower-grade, *IDH-*WT astrocytoma if molecular markers such as *TERT* promoter (*TERT*p) mutations, *EGFR* amplification (*EGFR*amp), or a chromosome 7 gain/chromosome 10 loss (+ 7/-10) signature are present, designated a molecular glioblastoma (MolGBM) [10, 12–16]. Presumably, these tumors represent GBM identified at slightly earlier stage in natural history, as a forme fruste before elaboration of the fully diagnostic histologic features found in the majority of cases.

Even though the 2021 WHO classification has outlined this framework, the impact of these molecular high-grade alterations in histologically lower-grade, *IDH-*WT astrocytoma has not been fully understood [17–21]. To date, studies comparing MolGBM with HistGBM have reported heterogeneous survival outcomes [17, 22, 23].

Therefore, our study aimed to systematically compare the prognosis of patients with surgically resected MolGBM with that of HistGBM-patients. Additionally, we investigated *IDH*-WT, *H3*-WT, *TERT*p-WT astrocytic tumors without genomic GBM-defining alterations to characterize their molecular profile and to assess whether all such tumors can be adequately classified within the current WHO 2021 framework or whether certain feature combinations remain difficult to categorize [24, 25]. Based on these findings, we aim to provide a more precise clinical prognostic assessment and identify potential implications for clinical management.

## Methods

### Study cohort

A total of 54 adult patients who underwent surgery for histologically lower-grade *IDH*-WT astrocytic tumors (WHO grade 2–3) at the University Hospital Carl Gustav Carus between 2008 and 2021 were included in our study. Patients who underwent biopsy only were excluded from the analysis to minimize potential confounding related to treatment heterogeneity and to ensure comparability of extent of resection (EOR) between the MolGBM and HistGBM cohorts.

Tumors were classified according to the 2021 WHO Classification of CNS tumors using a stepwise diagnostic workflow. First, cases harboring a non-canonical or occult *IDH*-R132-mutation (*n* = 7), as detected by *IDH1*-PCR, were excluded, leaving 47 tumor samples for further analysis.

Second, *TERT*p mutation analysis was performed. *IDH*-WT tumors harboring a *TERT*p mutation were classified as MolGBM, *IDH*-WT, CNS WHO grade 4 according to WHO 2021 criteria. Third, *IDH*-WT, *TERT*p-WT tumors underwent extended molecular characterization by whole-exome sequencing (WES) and DNA methylation profiling to assess additional GBM-defining alterations, including EGFRamp and combined chromosome 7 gain/10 loss. As *TERT*p-mutant tumors already fulfilled WHO 2021 molecular criteria for GBM, additional GBM-defining alterations such as EGFRamp or combined chromosome 7 gain/10 loss were not systematically assessed.

Among the cohort, 36 *IDH*-WT tumors harbored a *TERT*p-mutation, whereas 11 were *IDH*-WT, *TERT*p-WT. WES and methylation profiling revealed other genomic GBM-defining alterations in three *IDH*-WT, *TERT*p-WT tumors, including *EGFR*amp and + 7/-10 copy number changes, leading to their classification as MolGBM. Thus, in total, the MolGBM cohort comprised 39 patients.

The remaining eight tumors lacked genomic GBM-defining alterations and were retained as a descriptive group of histologically lower-grade *IDH*-WT, *TERT*p-WT astrocytic tumors without genomic GBM-defining alterations. *H3*-WT status (mainly *H3F3A/B mutations*) was determined by sequencing as part of the molecular analysis and was not used as an initial inclusion criterion. No *H3* alterations were detected. This group underwent further deep characterization by WES and methylation profiling.

The MolGBM cohort was compared with a cohort of 54 HistGBM patients diagnosed and surgically treated during the same institutional study period from 2008 to 2021. HistGBM was defined by histological WHO grade 4 features, including necrosis and/or microvascular proliferation.

The study was approved by the local Ethics Committee (EK 323122008), and all patients provided consent.

### Molecular analyses

*IDH1* and *TERT*p status were assessed, including analysis of the *IDH1* R132 region and the *TERT*p hotspot mutations C228T and C250T. *IDH*-WT, *TERT*p-WT tumors underwent WES and DNA methylation profiling. Additional methodological details are provided in the Supplementary Methods.

### Statistical analysis

Statistical analyses were performed using GraphPad Prism (v10.5.2). Group comparisons used Fisher’s exact or Mann-Whitney-U-tests. EOR was assessed on postoperative MRI and classified as gross total resection (GTR) when both contrast-enhancing and non-contrast-enhancing tumor had been completely removed, or as subtotal resection (STR) when residual contrast-enhancing or non-contrast-enhancing tumor was present. Complete Stupp treatment was defined as completion of concomitant radiochemotherapy followed by six cycles of adjuvant temozolomide; all other treatment courses were classified as incomplete Stupp treatment. Tumor volume was calculated for both MolGBM and HistGBM cohorts using the largest tumor dimensions with an ellipsoid approximation (V = π/6×L×W×H) [26, 27]. OS and progression-free survival (PFS) were estimated using the Kaplan-Meier method and compared using the log-rank test. OS was defined as the time from first surgery to death from any cause. PFS was defined as the time from first surgery to second surgery or, in the absence of re-operation, to radiographic progression. To adjust for potential confounding factors, multivariable Cox proportional hazards regression was performed. The final model included tumor group, age, EOR, and completion of the Stupp regimen. Details regarding treatment classification and model selection are provided in the Supplementary Methods. A two-sided p-value < 0.05 was considered statistically significant.

## Results

The *MolGBM cohort* consisted of 39 patients with *IDH*-WT tumors: 36 of whom harbored *TERT*p-mutations, three were identified via other genomic GBM-defining alterations in *TERT*p-WT tumors (*EGFR*amp (*n* = 1) or + 7/-10 scheme (*n* = 2)). The median age was 67.1 years (24.5–86.3), with 64.1% over 60 years; the male: female ratio was 1.6:1. Preoperative symptoms were notable for a high frequency of epileptic seizures (69.2%), with motor deficits (23.1%), and aphasia (23.1%) also noted. Within the MolGBM cohort, 84.6% (*n* = 33) were diagnosed WHO grade 3 and 15.4% (*n* = 6) were WHO grade 2.

The *HistGBM cohort* included 54 patients with *IDH*-WT tumors with histological features of GBM, with a median age of 60.2 years (22.9–79.8; 50% ≥60 years). The group comprised 32 men and 22 women (ratio 1.45:1). Motor deficits were the most frequent preoperative symptom (29.62%), followed by aphasia (25.9%) and headaches (24.1%). 

The descriptive group of histologically lower-grade *IDH*-WT, *TERT*p-WT astrocytic tumors without genomic GBM-defining alterations comprised eight patients. The median age at diagnosis was 58.4 years (47.5–74.2 years), with 50% ≥60 years, and the male: female ratio was 1:1. STR was performed in six cases and GTR in two. Tumors were in the frontal (*n* = 3), temporal lobes (*n* = 3) and the cerebellum (*n* = 2). Epileptic seizures were the most common symptom (62.5%). 

Patient characteristics for MolGBM and HistGBM are summarized in Table 1.

**Table 1:** Comparison of clinical, neurological, and molecular characteristics between MolGBM and HistGBM

Parameter	Total cohort (*n* = 93) / %	MolGBM (*n* = 39) / (median/%)	HistGBM (*n* = 54) / (median/%)	*p*-value
**Demographics**				
Age at diagnosis (years)	63.9 (22.85–86.31)	67.1 (24.53–86.31)	60.2 (22.85–79.76)	0.149
Age ≥ 60 years at diagnosis	*n* = 52 (55.9%)	*n* = 25 (64.1%)	*n* = 27 (50%)	0.207
Sex ratio (M: F)	56:37 1.51:1	24:15 1.6:1	32:22 1.45:1	0.999
**Therapy**				
STR	69 (74.2%)	29 (74.4%)	40 (74.1%)	0.999
GTR	24 (25.8%)	10 (25.6%)	14 (25.9%)	0.999
Biopsy	0	0	0	-
Adjuvant radiotherapy	54 (58.1%)	17 (43.6%)	37 (68.5%)	**0.02**
**Tumor Location**				
Frontal lobe	24 (25.8%)	6 (15.4%)	18 (33.3%)	0.058
Temporal lobe	19 (20.4%)	7 (17.9%)	12 (22.2%)	0.795
Parietal lobe	7 (7.5%)	4 (10.3%)	3 (5.6%)	0.447
Occipital lobe	2 (2.2%)	0 (0%)	2 (3.7%)	0.507
Insular	1 (1.1%)	1 (2.6%)	0 (0%)	0.419
Multilobar involvement	29 (31.2%)	16 (41%)	13 (24.1%)	0.112
Multifocal	11 (11.8%)	5 (12.8%)	6 (11.1%)	0.999
**Volume (cm** ^**3**^ **)**	-	17.4 (IQR: 7-50.5)	37.7 (IQR: 12.5–66.5)	0.273
**Preoperative Neurological Symptoms**				
Epileptic seizures	38 (40.8%)	27 (69.2%)	11 (20.4%)	< **0.0001**
Motor deficits	25 (26.9%)	9 (23.1%)	16 (29.6%)	0.636
Sensory deficits	11 (11.8%)	1 (2.6%)	10 (18.5%)	**0.0224**
Aphasia	23 (24.7%)	9 (23.1%)	14 (25.9%)	0.811
Visual disturbances	7 (7.5%)	2 (5.1%)	5 (9.3%)	0.695
Headaches	17 (18.3%)	4 (10.3%)	13 (24.1%)	0.108
**Postoperative Neurological Deficits**	25 (26.9%)	16 (41%)	9 (16.4%)	**0.0167**
**Proliferative Markers**				
Ki-67 index (median)	25%	10%	30%	**0.0001**
*MGMT* promoter	predominantly unmethylated	predominantly unmethylated	predominantly unmethylated	0.621
**Survival (median, months)**				
Overall survival	17.5	19.8	14.6	0.11
Progression-free survival	9.6	11.6	9.4	0.058

### Comparison of MolGBM and HistGBM

**Fig. 1:**
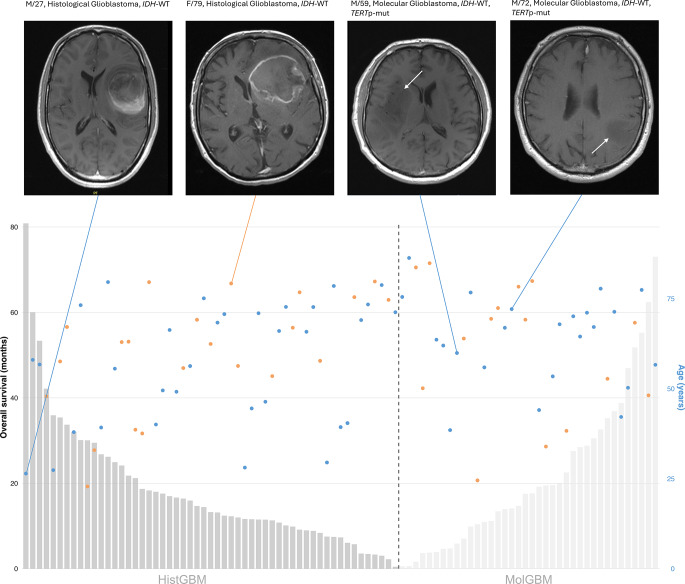
Comparison of HistGBM and MolGBM. Bars show overall survival (OS); dots indicate age at diagnosis and are color-coded by sex (blue for male, red for female). Representative contrast-enhanced T1-weighted MRI images are displayed above

#### Baseline characteristics

When comparing baseline characteristics between HistGBM and MolGBM, age distribution, sex ratio (Fig. 1), and EOR were broadly similar across cohorts. In both cohorts, STR was achieved in more than 70% of cases (HistGBM: *n* = 40; MolGBM: *n* = 29), whereas GTR was less frequent (HistGBM: *n* = 14; MolGBM: *n* = 10). Biopsy-only procedures were excluded according to the study design. Adjuvant therapy was dichotomized into complete (6 cycles) versus incomplete Stupp regimen (Supplementary Methods). HistGBM patients received complete Stupp therapy more frequently than MolGBM patients (68.5% vs. 43.6%; *p* = 0.02).

The frontal (25.8%) and temporal lobe (20.4%) were the most frequently affected areas. Approximately one-third (31.2%, *n* = 29) of tumors extended across multiple lobes. Multifocal tumor growth occurred in around 10% of cases. Only one tumor in the MolGBM cohort was infratentorial.

A comparison of the clinical and neurological presentations at diagnosis revealed substantial differences between the two cohorts. Epileptic seizures occurred in 27 patients (69.2%) in the MolGBM cohort, whereas only eleven patients (20.4%) in the HistGBM cohort were affected (*p* < 0.0001). Conversely, sensory deficits were rare in MolGBM (1/39) but more common in HistGBM (10/54, *p* = 0.0224).

During the postoperative follow-up, 16/39 MolGBM-patients exhibited the emergence of new neurological deficits, in comparison to only 9/54 HistGBM-patients (*p* = 0.016).

#### Tumor volume and Ki-67

The median tumor volume was lower in MolGBM than in HistGBM (17.4 vs. 37.7 cm³), without reaching statistical significance (*p* = 0.273).

HistGBM exhibited a higher Ki-67 proliferation index (30%) than MolGBM (10%; *p* = 0.0001). *MGMT* promoter (*MGMT*p) status was assessable in 25/39 MolGBM and 45/54 HistGBM cases, with unmethylated status in 14 and 28 cases, respectively.

#### Survival analysis

**Fig. 2:**
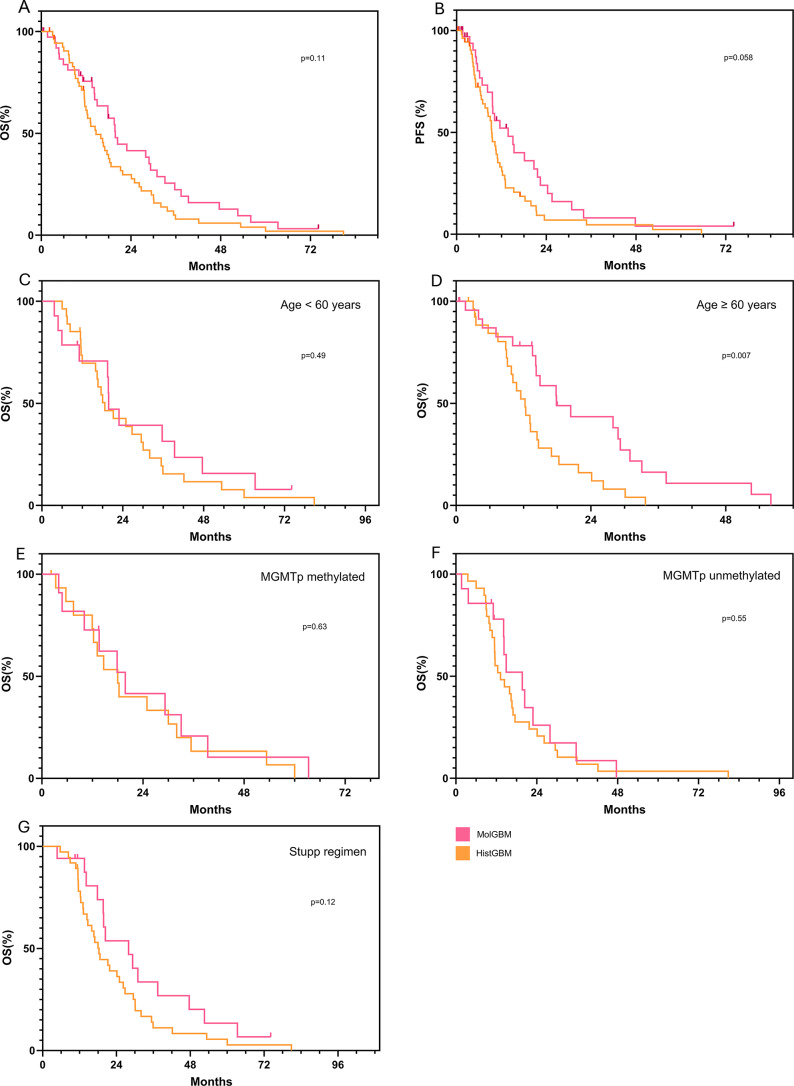
Kaplan-Meier curves for overall survival (OS; **A**) and progression-free survival (PFS; **B**) in HistGBM (orange) and MolGBM (pink). Kaplan-Meier analyses of overall survival comparing HistGBM and MolGBM, stratified by age ((**C**) < 60 years, (**D**) ≥ 60 years), *MGMT* promoter methylation status ((**E**) methylated, (**F**) unmethylated), and treatment ((**G**) patients treated with Stupp regimen)). Group comparisons were performed using the log-rank test

Median OS was 19.8 months in MolGBM compared with 14.6 months in HistGBM (*p* = 0.11, Fig. 2). Median PFS was 11.6 months in MolGBM and 9.4 months in HistGBM (*p* = 0.058). 

Stratified Kaplan-Meier analyses for OS were performed by age, *MGMT*p methylation status and treatment.

Patients were dichotomized at 60 years. No difference between HistGBM and MolGBM was observed in patients < 60 years (median OS: 18.7 vs. 19.8 months; *p* = 0.49), whereas patients ≥ 60 years showed significantly longer OS in the MolGBM cohort (17.9 months in MolGBM vs. 12.3 months in HistGBM; *p* = 0.0079).

*MGMT*p methylation status did not affect OS differences between HistGBM and MolGBM in either methylated (18 vs. 19.8 months; *p* = 0.63) or unmethylated tumors (13.2 vs. 19.7 months; *p* = 0.55).

Among patients receiving radiochemotherapy, MolGBM showed numerically longer OS (27.9 months in MolGBM vs. 18.3 months in HistGBM; *p* = 0.124).

To account for potential confounding by clinical and treatment-related factors, multivariable Cox proportional hazards regression was performed. In the final adjusted model including tumor group, age, EOR, and completion of the Stupp regimen, MolGBM was independently associated with more favorable OS compared with HistGBM (HR 0.40, 95% CI 0.24–0.67, *p* = 0.0005, Supplementary Table 1 and 2).

### Comparison of *IDH*-WT, *H3*-WT, *TERT*p-WT astrocytic tumors without genomic GBM-defining alterations versus the total GBM cohort

In an exploratory descriptive comparison, the eight patients with *IDH*-WT, *H3*-WT, *TERT*p-WT astrocytic tumors without genomic GBM-defining alterations were compared with the total GBM cohort (HistGBM+MolGBM). This comparison was performed to characterize the clinical behavior of tumors that did not fulfill molecular or histological criteria for GBM according to the 2021 WHO classification.

The median OS was significantly longer in this descriptive subgroup at 63.4 months compared with 17.5 months in the total GBM cohort (*p* = 0.0006). Similarly, median PFS was longer in this subgroup compared with the total GBM cohort (39.1 vs. 9.6 months; *p* = 0.0073). The median Ki-67 proliferation index was lower in these tumors than in the total GBM cohort (10% vs. 25%; *p* = 0.0123).

Infratentorial tumor location was more frequent among histologically lower-grade *IDH*-WT, *H3*-WT, *TERT*p-WT astrocytic tumors without genomic GBM-defining alterations than in the total GBM cohort (2/8, 25% vs. 1/93, 1.1%; *p* = 0.0157). Given the small number of cases and the marked molecular heterogeneity identified by extended molecular characterization, these findings should be interpreted descriptively and not as evidence of a distinct favorable tumor entity.

### Molecular characterization of *IDH*-WT, *H3*-WT, *TERT*p-WT astrocytic tumors without genomic GBM-defining alterations

**Table 2:** Patients´ and tumor characteristics

Patient	Age (years)	Sex	Tumor location	WHO grade	Histologic features	Genomic alterations	Final Diagnosis
DD1	67.7	M	cerebellar	3	Pilocytic astrocytoma–like growth pattern with increased proliferative activity, without Rosenthal fibers	*NF1* and *ATRX* mutations *CDKN2A/B* deletion	High-grade astrocytoma with piloid features (HGAP)
DD2	47.5	F	frontal	2	Unusual glial tumor, predominantly oligodendroglial but also showing astrocytic components	*ATRX* mutation	Ependymal tumor, classifier score < 0.333
DD3	48.7	M	cerebellar	3	Hypercellular tumor with predominantly astrocytic differentiation; occasional protein droplets present	*NF1* and *ATRX* mutations *CDKN2A/B* deletion	High-grade astrocytoma with piloid features (HGAP)
DD4	50.3	F	temporal	3	Focally hypercellular, predominantly oligodendroglial; focally loose astrocytic areas with increased fibrillary matrix	-	Oligodendroglioma, *IDH*-mut, 1p/19q, classifier score 0.99 (no *IDH*-mutation detected)
DD5	71.2	M	temporal	3	glioma, most consistent with an anaplastic oligodendroglioma	*PDGFRA* amplification	Diffuse pediatric-type high-grade glioma, *H3*- and *IDH-*wild-type
DD6	66.1	F	frontal	3	diffuse glioma with focal anaplasia	*ATRX* and *NTRK1* mutations	Ependymal tumor, classifier score 0.452
DD7	50.1	M	frontal	3	diffuse infiltrating glial tumor	*NTRK1* and *SETD2* mutations *NF1* deletion	Diffuse glioneuronal tumor with oligodendroglioma-like features and nuclear clusters (DGONC)
DD8	74.2	F	temporal	2	diffusely infiltrating, astrocytically differentiated glioma	-	No definitive methylation-based classification, score < 0.3

In this cohort, *ATRX* (50%, *n* = 4) and *NF1* (*n* = 3) were frequent events.

Two *IDH*-WT, *H3*-WT, *TERT*p-WT tumors (DD1/DD3) exhibited a molecular profile consistent with high-grade astrocytoma with piloid features (HGAP), characterized by mutations in *NF1* and *ATRX* and homozygous deletions of *CDKN2A*/*B* [28, 29]. These cerebellar tumors displayed piloid-like architecture and showed elevated proliferation. Radiological assessment and histology lacked GBM-defining features, aligning with the WHO 2021 criteria for HGAP.

Another *IDH*-WT, *H3*-WT, *TERT*p-WT tumor (DD5) in a 71-year-old patient demonstrated alterations in *PDGFRA*. Despite this elevated age, methylation profiling resulted in a classifier score of 0.639 for diffuse pediatric-type high-grade glioma, *H3*- and *IDH*-WT, indicating limited confidence. Nevertheless, the *IDH*-WT, *PDGFRA* gain, and a high-grade morphology were compatible with this entity [30].

A further *IDH*-WT, *H3*-WT, *TERT*p-WT tumor (DD7) exhibited *SETD2-* and *NTRK1*-mutations. Methylation profiling revealed the highest similarity to the epigenetic class of diffuse glioneuronal tumor with oligodendroglioma-like features and nuclear clusters (DGONC) [31].

For four *IDH*-WT, *H3*-WT, *TERT*p-WT tumors, classification remained inconclusive (Supplementary Information). These tumors shared low proliferative activity, absence of necrosis or microvascular proliferation, and lacked hallmark genomic alterations typically used to delineate diffuse glioma subtypes. One case (DD8) showed no classifier match after DNA methylation analysis. Two cases (DD2/DD6) demonstrated only a low-confidence classifier score of 0.33 and 0.452 for ependymal tumors. In another case (DD4), a high classifier score (0.99) suggested oligodendroglioma, *IDH*-mutant; however, this was not supported by molecular analysis, as no *IDH1/2*-mutation was detected by Sanger sequencing or WES. We cannot exclude loss of the *IDH*-mutation, which has been reported in rare cases of late-stage glioma [32].

**Fig. 3:**
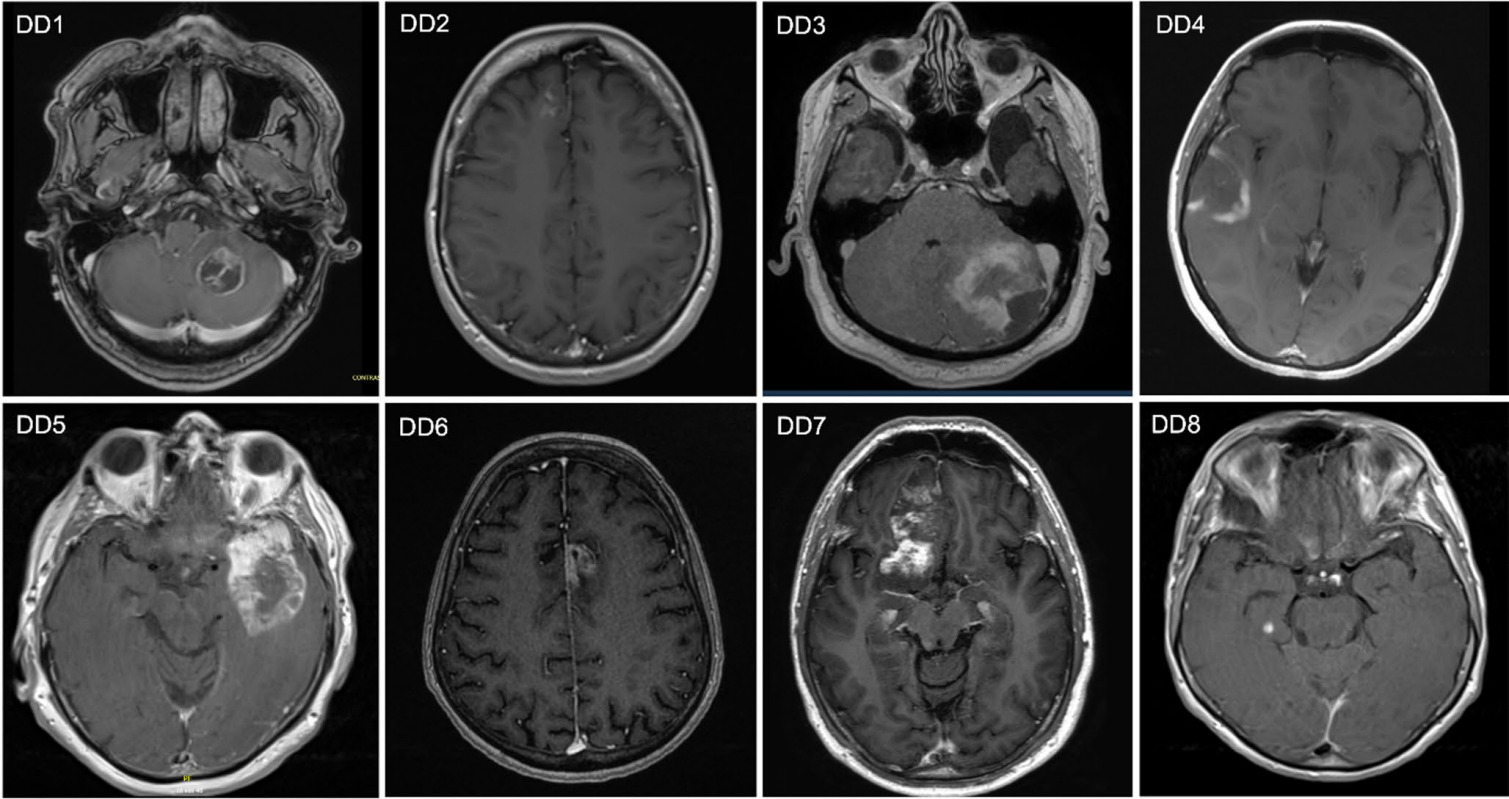
Contrast-enhanced T1-weighted MRI of *IDH*-WT, *H3*-WT, *TERT*p-WT astrocytic tumors without genomic GBM-defining alterations (*n* = 8)

Patients´ characteristics are summarized in Table 2 and Supplementary Fig. 1. MRI findings across tumors are shown in Fig. 3.

## Discussion

Molecular profiling has become increasingly central to glioma classification [33–36]. Categorization of glioma based on molecular genetic features has identified biologically distinct subgroups associated with different survival outcomes [7, 37]. *IDH*-WT astrocytoma harboring *TERT*p-mutations, *EGFR*amp, or + 7/–10 CNVs are being considered as GBM according to the 2021 WHO classification of CNS tumors, even when histologically lower-grade [10, 13], although with potential alternative diagnoses possible [21].

We compared surgically resected MolGBM with HistGBM and observed clinically relevant differences, including higher frequency of epilepsy at initial presentation in MolGBM-patients, lower Ki-67 proliferation indices, differences in postoperative neurological deficits, and treatment patterns. These findings support the clinical heterogeneity of molecularly defined *IDH*-WT diffuse glioma. *IDH*-WT, *H3*-WT, *TERT*p-WT astrocytic tumors without genomic GBM-defining alterations showed distinct clinical courses and molecular profiles.

### Clinical characteristics

The MolGBM cohort comprised 36 histologically lower-grade, *IDH*-WT, *TERT*p-mutant glioma and three *IDH*-WT, *TERT*p-WT tumors with additional genomic GBM-defining alterations.

Previous studies have suggested that MolGBM may occur more frequently in younger patients compared to HistGBM. Guo et al. reported that patients with MolGBM were approximately six years younger than those with HistGBM, while Lee et al. observed a mean age difference of about four years [17, 23]. However, in our series, we observed no difference in the age distribution of MolGBM- and HistGBM-patients. Systematic data on the clinical presentation of MolGBM remain limited [17], as most studies have primarily focused on molecular stratification and survival outcomes [23, 39]. In line with Guo et al. [17], epileptic seizures in our series occurred significantly more frequent in MolGBM-patients. This finding may suggest that seizure-related presentation contributes to earlier clinical detection of MolGBM, at a stage when tumors may still lack histological grade 4 features. However, seizures should not be interpreted as evidence of an earlier biological disease stage *per se.* In addition, despite comparable tumor volumes, anatomical distribution, and EOR, MolGBM-patients experienced a higher rate of new postoperative neurological deficits. This observation likely supports the hypothesis that histological lower-grade glioma harboring genomic GBM-defining alterations may present with a biologically more diffuse and infiltrative growth pattern than HistGBM [10, 13, 40, 41], which could underlie functional vulnerability even in tumors lacking histological hallmarks of GBM [42]. From a clinical perspective, these findings demonstrate that tumor biology correlates with a certain clinical phenotype [43].

### Survival outcomes

Several studies have examined survival differences between MolGBM and HistGBM.

However, some of these studies have potential confounding by differences in EOR or by the inclusion of biopsy-only patients, biasing the reported survival outcomes.

In a study by Lee et al., survival was significantly longer in patients with MolGBM compared with HistGBM, with a median OS of 30.2 versus 18.4 months (*p* = 0.001). In this cohort of 983 patients (MolGBM: *n* = 52; HistGBM: *n* = 931), the EOR differed significantly between groups, with approximately 25% of the MolGBM-patients having received biopsy only.

In contrast, Ramos-Fresnedo et al. reported no difference in OS between MolGBM (26 months) and HistGBM (21 months) in a cohort of 708 patients, including 65 MolGBM cases. In this study, biopsy-only procedures were performed in approximately 50% of MolGBM-patients, which the authors identified as a source of bias, as limited tissue sampling may lead to histological undersampling and misclassification of grade 4 tumors as lower-grade lesions [39]. In line with the latter findings, in a prospective study by Zhang et al. 38 patients (surgery (*n* = 21) or biopsy (*n* = 17)) with *IDH*-WT diffuse astrocytic tumors with genomic GBM-defining alterations received aggressive therapy following molecular diagnosis, achieving a median OS of 23.8 months.

To minimize these confounding factors, we excluded patients who underwent biopsy alone and ensured that the distribution of the EOR was comparable between MolGBM and HistGBM.

In our study, no statistically significant difference in OS was observed between MolGBM and HistGBM in unstratified survival analysis, consistent with findings in previous studies.

Beyond unstratified OS analyses, Patil et al. performed subgroup-based analyses and reported improved survival for *MGMT*p-unmethylated MolGBM (OS 15.2 months) compared with *MGMT*p-unmethylated HistGBM (12.7 months) [22]. Following this approach, we conducted predefined subgroup analyses stratified by *MGMT*p methylation status, age, and treatment. In our cohort, both MolGBM and HistGBM were characterized by a predominance of *MGMT*p-unmethylation [44]. However, in contrast to the study by Patil et al., we did not observe a survival difference between MolGBM and HistGBM in either *MGMT*p-methylated or *MGMT*p-unmethylated subgroups.

Moreover, exploratory predefined subgroup analyses by age in our study revealed significant survival advantages for MolGBM patients aged 60 years or older, whereas survival was largely comparable between MolGBM and HistGBM in patients < 60 years. This finding is consistent with the generally more favorable prognosis of younger patients irrespective of tumor entity. Of course, it should be considered that in younger patients, particularly when lower-grade tumor features are suspected/seen, surgical treatment tends to be more extensive, which may partly explain their better outcomes. In patients over 60, treatment is generally less intensive, and OS is worse; however, MolGBM-patients still show a better OS than HistGBM-patients in the same subgroup, suggesting a potential window of therapeutic responsiveness may exist in this cohort.

Indeed, outcomes for MolGBM can be further improved under more intensive treatment. Among patients treated with radiochemotherapy, MolGBM showed numerically longer OS (27 months) compared to HistGBM, although statistical significance was not reached. In our study, MolGBM-patients were less frequently treated with a complete Stupp protocol, likely reflecting their initial classification as lower-grade astrocytoma and, in part, the older age of a subset of patients, which may have limited the treatment with an aggressive radiotherapy or resulted in radiotherapy alone. Because treatment intensity is closely linked to survival, unadjusted survival comparisons may be biased. We therefore performed multivariable Cox regression analysis adjusting for clinically relevant variables. In the final model including tumor group, age, EOR, and completion of the Stupp regimen, being in the MolGBM cohort was an independent factor for a more favorable OS compared with HistGBM. This finding does not imply that MolGBM represents a uniformly favorable entity, but rather suggests that molecularly defined *IDH*-WT diffuse glioma are clinically heterogeneous and that survival differences are influenced by treatment-related and clinical confounders.

The interpretation of our MolGBM cohort is also relevant in light of cIMPACT-NOW update 11 [21]. Under the WHO 2021 framework, detection of a *TERT*p mutation in an *IDH*-WT diffuse astrocytic tumor can be sufficient for classification as glioblastoma, *IDH*-WT, CNS WHO grade 4, without necessarily prompting comprehensive molecular characterization in every case [10]. Accordingly, most MolGBM cases in our cohort were classified based on *IDH*-WT status and *TERT*p mutation. Although our analysis was retrospective, in a prospective real-world diagnostic setting this classification would generally support glioblastoma-directed management, including radiochemotherapy according to the Stupp regimen. Since additional GBM-defining alterations were not systematically assessed in all *TERT*p-mutant tumors, these cases should be regarded as *TERT*p-defined MolGBM rather than as tumors proven to harbor *TERT*p mutation as the only GBM-defining alteration. This distinction is important because cIMPACT-NOW update 11 recommends caution when assigning CNS WHO grade 4 to histologically lower-grade *IDH*-WT diffuse glioma solely on the basis of *TERT*p mutation [21]. Our findings are compatible with this cautionary perspective. The more favorable adjusted outcome observed in MolGBM suggests that *TERT*p-defined, histologically lower-grade *IDH*-WT diffuse glioma may not be fully equivalent to HistGBM in all cases. At the same time, survival in the MolGBM cohort remained poor, supporting the clinical relevance of identifying these tumors as aggressive *IDH*-WT diffuse glioma requiring close clinical attention.

This point also highlights a strength of the present study: our cohort reflects a real-world diagnostic scenario under the WHO 2021 framework, in which histologically lower-grade *IDH*-WT tumors were often classified as GBM once a *TERT*p mutation was detected, without comprehensive molecular characterization in every case. Thus, our data provide clinically relevant information on the *TERT*p-defined MolGBM subgroup now being reconsidered in light of cIMPACT-NOW update 11. The more favorable adjusted outcome and clinical differences observed in MolGBM support the view that these tumors are heterogeneous and may not be fully equivalent to HistGBM in all cases. Until future WHO classifications incorporate these refinements, WHO 2021 remains the applicable diagnostic framework, while cIMPACT-NOW update 11 provides a transitional perspective emphasizing critical re-evaluation and broader molecular characterization of *TERT*p-defined, histologically lower-grade *IDH*-WT tumors whenever feasible.

### *IDH*-WT, *H3*-WT, *TERT*p-WT astrocytic tumors without genomic GBM-defining alterations

In this cohort, these eight tumors demonstrated significantly longer OS and PFS compared with the combined *IDH*-WT GBM cohort, but this observation should be interpreted descriptively due to the small number of cases and their molecular heterogeneity.

An important question was whether these tumors could be adequately classified within the refined WHO 2021 framework. Rather than representing a single entity, these tumors form a basket of different molecularly distinct entities. Extended molecular characterization identified cases suggestive of HGAP, diffuse pediatric-type high-grade glioma, *H3*- and *IDH*-WT, and DGONC. However, despite WES and DNA methylation profiling, four tumors could not be confidently assigned to any recognized WHO 2021 category.

In the current study, *ATRX*- and *NF1* alterations were frequently observed, in line with previous reports. Williams et al. reported *ATRX*-mutations in 37.5% of patients with *IDH*-WT, *TERT*p-WT GBM, a significantly higher frequency than observed in *IDH*-WT, *TERT*p-mutant GBM (6.5%) [45]. Similarly, Diplas et al. found *ATRX*-mutations in 20% and *NF1* alterations in 24% of all *IDH*-WT, *TERT*p-WT GBM [35].

These findings emphasize that *IDH*-WT, *TERT*p-WT lower-grade astrocytic tumors without canonical genomic GBM-defining alterations require integrated histopathological, molecular, epigenetic, and radiological assessment and should not be treated as a homogeneous prognostic group.

### Limitations

Several limitations should be acknowledged. This retrospective single-center study included a limited number of patients, reducing statistical power, particularly for subgroup analyses. Although biopsy-only cases were excluded and EOR was comparable, treatment imbalance remained a relevant confounder; despite multivariable adjustment, residual confounding cannot be excluded. Diagnostic workflows evolved during the long study period from 2008 to 2021, and comprehensive molecular profiling, including WES and DNA methylation profiling, was not uniformly available for all tumors. Finally, subgroup and sensitivity analyses, including age-stratified analyses, were exploratory and should be interpreted cautiously given the limited sample size.

## Conclusions

In summary, while MolGBM and HistGBM showed comparable OS, MolGBM was associated with more favorable survival after adjustment for clinical and treatment-related confounders. Beyond survival, MolGBM differed clinically from HistGBM by a higher frequency of epileptic seizures at presentation. These findings support the clinical and biological heterogeneity of molecularly defined *IDH*-WT diffuse glioma, particularly among *TERT*p-defined MolGBM.

Until future WHO classifications formally incorporate the refinements proposed by cIMPACT-NOW update 11, WHO 2021 and cIMPACT-NOW update 11 should be viewed as complementary rather than contradictory: WHO 2021 remains the currently applicable diagnostic framework, while cIMPACT-NOW update 11 underscores the need to critically re-evaluate histologically lower-grade *IDH*-WT tumors classified as glioblastoma primarily on the basis of *TERT*p mutation. Histologically lower-grade *IDH*-WT, *TERT*p-WT astrocytic tumors without genomic GBM-defining alterations likewise showed marked diagnostic heterogeneity and require comprehensive molecular characterization.

## Electronic Supplementary Material

Below is the link to the electronic supplementary material.

Supplementary Material 1

Supplementary Material 2

Supplementary Material 3

Supplementary Material 4

## Data Availability

No datasets were generated or analysed during the current study.
