# Supplementary material for: Comparison between molecular and histological IDH-wild-type glioblastoma and extensive subgroup analysis of IDH-wild-type astrocytic tumors without genomic glioblastoma-defining alterations
Source: J Neurooncol. 2026 Jun 10;178(2):58. doi: 10.1007/s11060-026-05637-w (PMC13253596; doi:10.1007/s11060-026-05637-w)
Supplement: Supplementary file 1 — Supplementary Material 1 [file 11060_2026_5637_MOESM1_ESM.docx]

**Supplementary Information**

Comparison between molecular and histological *IDH*-wild-type glioblastoma and extensive subgroup analysis of *IDH*-wild-type astrocytic tumors without molecular glioblastoma-defining alterations

Anna M. Seifert^1^, Sven Richter^1,2^, André Sagerer^1^, Ioana Lemnian^3^, Sylvia Herold^4^, Sascha Brückmann^4^, Dimitrios Emmanouilidis^1^, Majd Alkhatib^1^, Ilker Y. Eyüpoglu^1^, Erik A. Williams^5,6^, Daniel P. Cahill^7,8^, Tareq A. Juratli^1,7,9^

1 Department of Neurosurgery, Faculty of Medicine and University Hospital Carl Gustav Carus, TUD Dresden University of Technology, Fetscherstraße 74, 01307 Dresden, Germany

2 Else Kröner Fresenius Center for Digital Health, Faculty of Medicine, TUD Dresden University of Technology, Dresden, Germany

3 PathoNext GmbH, Molecular Pathology, Leipzig, Germany

4 Department of Pathology, Faculty of Medicine and University Hospital Carl Gustav Carus, TUD Dresden University of Technology, Fetscherstraße 74, 01307 Dresden, Germany

5 Foundation Medicine Inc, Cambridge, MA, USA

6 Department of Pathology and Laboratory Medicine, University of Miami, Sylvester Comprehensive Cancer Center, Miami, FL, USA

7 Laboratory of Translational Neuro-Oncology, Department of Neurosurgery, Massachusetts General Hospital, Harvard Medical School, Boston, MA, USA

8 Department of Neurosurgery, Massachusetts General Hospital, Harvard Medical School, Boston, MA, USA

9 National Center for Tumor Diseases (NCT), NCT/UCC Dresden, a partnership between DKFZ, Faculty of Medicine and University Hospital Carl Gustav Carus, TUD Dresden University of Technology, and Helmholtz-Zentrum Dresden-Rossendorf (HZDR), Dresden, Germany

Corresponding author:

Tareq A. Juratli, MD

Department of Neurosurgery, University Hospital Carl Gustav Carus, TU Dresden, Germany

Email: Tareq.Juratli@ukdd.de

**Supplementary Methods**

**Definition Stupp treatment:**

Complete Stupp treatment was defined as completion of radiotherapy with concomitant daily temozolomide at 75 mg/m² followed by six cycles of adjuvant temozolomide at 150–200 mg/m² on days 1–5 of each 28-day cycle. Radiotherapy alone, no adjuvant therapy, fewer than six cycles of adjuvant temozolomide, or premature discontinuation of concomitant or adjuvant treatment were classified as incomplete Stupp treatment.

**PCR/Sanger Sequencing:**

**Isolation of Genomic DNA from Preserved Tumor Tissue and DNA Quantification**

Genomic DNA was extracted from frozen tumor tissue using the QIAamp DNA Mini Kit (QIAGEN) following the manufacturer´s instructions. Approximately 25 mg of tissue was lysed overnight with Proteinase K in ATL buffer at 56 °C. DNA was eluted in AE buffer, yielding concentrations between 25 and 75 ng/µl. DNA concentration and purity were assessed photometrically using a BioTek microplate reader and Gen5 software, with absorbance measured at 260, 280, and 320 nm. Purity was assessed by the 260/280 ratio (optimal 1.8 and 2.0).

***MGMT* promoter methylation status**

*MGMT* promoter methylation status was assessed by pyrosequencing as part of routine clinical molecular diagnostics. Tumors with *MGMT* promoter methylation levels ≥8% were classified as methylated, whereas tumors with methylation levels <8% were classified as unmethylated.

**Polymerase Chain Reaction (PCR) and Sanger Sequencing of *IDH1* and *TERT*p**

*IDH1* R132H status was initially assessed by immunohistochemistry in all cases (n=54). All tumors were subsequently analyzed by Sanger sequencing of the *IDH1* R132 region. Tumors classified as *IDH1*-WT (n=47) underwent additional Sanger sequencing of the *TERT*p hotspot mutations (C228T and C250T).

Screening for *IDH1* and *TERT*p mutations was performed by PCR amplification using validated primers (Eurofins Genomics). Primer sequences were as follows: *IDH1* forward 5′-CGG TCT TCA GAG AAG CCA TT-3′ and reverse 5′-GCA AAA TCA CAT TAT TGC CAA C-3′; *TERT*p forward 5′-CTC CTG CCC CTT CAC CTT-3′ and reverse 5′-CAG CGC TGC CTG AAA CTC-3′.

PCR amplification and mutation detection followed previously established protocols. Amplicons were subjected to Sanger sequencing (Microsynth Seqlab GmbH, Göttingen), and chromatograms were analyzed using ApE software.

Ambiguous *TERT*p PCR results and all *TERT*p-WT tumors were subsequently subjected to whole-exome sequencing (WES) and methylation profiling for comprehensive molecular classification. In this subgroup, both *IDH1* and *IDH2* mutation status were further evaluated. **Accordingly, *IDH2* mutation status was available only for tumors analyzed by WES.**

**WES**

**DNA Isolation, Quality Control, and Sample Selection**

Genomic DNA was extracted from fresh frozen tissue of tumor samples classified as *IDH*-wild-type and *TERTp*-wild-type using **Maxwell RSC genomic DNA Kit** (Promega, USA). DNA quantity and integrity was assessed via **Fluorometry** (Qubit, ThermoFisher, USA) and TapeStation using the **Genomic DNA Screen Tape Assay** (Agilent Technologies, USA), respectively.

**Whole Exome Sequencing and Data Analysis**

DNA libraries were prepared from the genomic DNA using the **Twist Library Preparation EF Kit 2.0 with UDI Adapter System** (Twist, USA)**.** Target Enrichment was performed with the panel **Twist Exom 2.0 plus Comprehensive Exome spike-in** in combination  with  **Twist Standard Hyb and Wash Kit v2** (Twist, USA).

Enrichment pools are sequenced using the **Illumina NovaSeq 6000 platform** with **paired-end 150 bp reads, v1.5 reagents (Illumina, USA)**.

The data analysis has been performed using the IVDR certified software varvis® 2.4.1 (Limbus, Rostock), which includes data quality control, mapping to the human genome hg38, variant calling and copy number variation for each sample, as well as assesment of multiple in-silico prediction scores of the variant effect, prevalence in the general population (gnomAD v4.0), and presence in ClinVar.

**Variant filtering**

In order to pinpoint additional diagnostically relevant molecular alterations, a virtual panel corresponding to the list of genes included in the TruSight Oncology 500 Panel (Illumina) has been applied. In this way the variants present in more than 500 cancer-associated genes, including *H3 (including H3 K27M and H3 G34 alterations)*, *NF1*, *EGFR*, *ATRX*, *POLR2A*, *PIK3CA*, *NOTCH3*, and *NTRK1* have been selected.

Further filtering has been performed to ensure high quality of the variants (Qual-Index>100), a high/moderate impact on the protein by omitting synonymous variants, and an allele frequency in the general population (gnomAD) below 0.005. Variants that have been reported frequently in the PathoNext internal variant database have been removed.

**Methylation analysis**

**Tissue preparation and methylation analysis (including MGMT-locus)**

Methylation analysis was performed on formalin-fixed paraffin-embedded (FFPE) tumor tissues. For this purpose, tumor cell content was evaluated using H&E slides and respective tissue areas were dissected and the DNA was extracted using QiaAmp DNA Micro Kit (QIAGEN, Hilden, Germany) following the manufacturer’s instructions. 250 ng of DNA was used for bisulfite conversion using the EZ DNA Lightning Methylation-Kit (Zymo Research), followed by FFPE restoration using Inﬁnium HD FFPE DNA Restore Kit (Illumina) according to the protocol. Samples were further processed on the Inf MethylationEPIC V2.0 array following the manufacturer’s instructions and scanned using Illumina NextSeq550.

Resulting .idat-files were used for calculation of the “classifier score” (version 12.8, <https://app.epignostix.com>) as well as copy-number profiles using a reference data base, which can be accessed at <https://www.molecularneuropathology.org>. A good match with the underlying reference group can be found based on an associated calibrated score of ≥0.9 whereas scores 0.5-0.9 are not a match per se and require a histomorphological re-evaluation.
