# Supplementary material for: Comparison between molecular and histological IDH-wild-type glioblastoma and extensive subgroup analysis of IDH-wild-type astrocytic tumors without genomic glioblastoma-defining alterations
Source: J Neurooncol. 2026 Jun 10;178(2):58. doi: 10.1007/s11060-026-05637-w (PMC13253596; doi:10.1007/s11060-026-05637-w)
Supplement: Supplementary file 2 — Supplementary Material 2 [file 11060_2026_5637_MOESM2_ESM.docx]

**Supplementary Information**

Comparison between molecular and histological *IDH*-wild-type glioblastoma and extensive subgroup analysis of *IDH*-wild-type astrocytic tumors without molecular glioblastoma-defining alterations

Anna M. Seifert^1^, Sven Richter^1,2^, André Sagerer^1^, Ioana Lemnian^3^, Sylvia Herold^4^, Sascha Brückmann^4^, Dimitrios Emmanouilidis^1^, Majd Alkhatib^1^, Ilker Y. Eyüpoglu^1^, Erik A. Williams^5,6^, Daniel P. Cahill^7,8^, Tareq A. Juratli^1,7,9^

1 Department of Neurosurgery, Faculty of Medicine and University Hospital Carl Gustav Carus, TUD Dresden University of Technology, Fetscherstraße 74, 01307 Dresden, Germany

2 Else Kröner Fresenius Center for Digital Health, Faculty of Medicine, TUD Dresden University of Technology, Dresden, Germany

3 PathoNext GmbH, Molecular Pathology, Leipzig, Germany

4 Department of Pathology, Faculty of Medicine and University Hospital Carl Gustav Carus, TUD Dresden University of Technology, Fetscherstraße 74, 01307 Dresden, Germany

5 Foundation Medicine Inc, Cambridge, MA, USA

6 Department of Pathology and Laboratory Medicine, University of Miami, Sylvester Comprehensive Cancer Center, Miami, FL, USA

7 Laboratory of Translational Neuro-Oncology, Department of Neurosurgery, Massachusetts General Hospital, Harvard Medical School, Boston, MA, USA

8 Department of Neurosurgery, Massachusetts General Hospital, Harvard Medical School, Boston, MA, USA

9 National Center for Tumor Diseases (NCT), NCT/UCC Dresden, a partnership between DKFZ, Faculty of Medicine and University Hospital Carl Gustav Carus, TUD Dresden University of Technology, and Helmholtz-Zentrum Dresden-Rossendorf (HZDR), Dresden, Germany

Corresponding author:

Tareq A. Juratli, MD

Department of Neurosurgery, University Hospital Carl Gustav Carus, TU Dresden, Germany

Email: Tareq.Juratli@ukdd.de

**Supplementary Table 1. Multivariable Cox regression analysis for overall survival**

Cox proportional hazards regression was performed using overall survival (OS) as the time variable and death as the event variable. The model included tumor group, age, extent of resection (EOR), and completion of adjuvant therapy. Hazard ratios below 1 indicate a reduced hazard of death. Confidence intervals were calculated using profile likelihood. Although the univariable OS comparison between MolGBM and HistGBM was not statistically significant in the overall cohort, MolGBM was independently associated with improved OS after adjustment for established prognostic factors.

| **Variable** | **Coding/comparison** | **β coefficient** | **Hazard ratio** | **95% CI** | **Likelihood ratio** | **p value** | **VIF** |
| --- | --- | --- | --- | --- | --- | --- | --- |
| Group: MolGBM | MolGBM vs HistGBM | -0.911 | 0.40 | 0.24–0.67 | 12.29 | 0.0005 | 1.81 |
| Age | Per additional year | 0.018 | 1.02 | 1.00–1.03 | 5.79 | 0.0161 | 3.31 |
| GTR | EOR[GTR] vs EOR[STR] | -1.021 | 0.36 | 0.21–0.60 | 16.38 | <0.0001 | 1.37 |
| Adjuvant therapy [complete Stupp regimen] | Complete vs incomplete/no complete Stupp regimen | -1.071 | 0.34 | 0.20–0.58 | 15.80 | <0.0001 | 2.01 |

*Abbreviations: CI, confidence interval; EOR, extent of resection; GBM, glioblastoma; HistGBM, histological glioblastoma; MolGBM, molecular glioblastoma; OS, overall survival; VIF, variance inflation factor.*

**Supplementary Table 2. Model diagnostics**

The final model included 93 observations and 83 events, corresponding to 20.75 events per parameter estimate. Variance inflation factors ranged from 1.37 to 3.31, indicating no relevant multicollinearity among the included covariates.

| **Parameter** | **Value** |
| --- | --- |
| Observations included | 93 |
| Rows skipped due to missing data | 0 |
| Deaths/events | 83 |
| Censored observations | 10 |
| Number of covariates/parameter estimates | 4 |
| Events per parameter estimate | 20.75 |
| AIC, null model | 585.8 |
| AIC, selected model | 556.6 |
| Partial log likelihood, selected model | -274.3 |
| Likelihood ratio test | χ² = 37.17, p < 0.0001 |
| Wald test | χ² = 33.19, p < 0.0001 |
| Score test | χ² = 34.76, p < 0.0001 |
| Maximum VIF | 3.31 |
