# Supplementary material for: Comparison between molecular and histological IDH-wild-type glioblastoma and extensive subgroup analysis of IDH-wild-type astrocytic tumors without genomic glioblastoma-defining alterations
Source: J Neurooncol. 2026 Jun 10;178(2):58. doi: 10.1007/s11060-026-05637-w (PMC13253596; doi:10.1007/s11060-026-05637-w)
Supplement: Supplementary file 3 — Supplementary Material 3 [file 11060_2026_5637_MOESM3_ESM.pdf]

Supplementary Information

Comparison between molecular and histological *IDH*-wild-type glioblastoma and extensive subgroup analysis of *IDH*-wild-type astrocytic tumors without molecular glioblastoma-defining alterations

Anna M. Seifert[1], Sven Richter[1,2], André Sagerer[1], Ioana Lemnian[3], Sylvia Herold[4], Sascha Brückmann[4], Dimitrios Emmanouilidis[1], Majd Alkhatib[1], Ilker Y. Eyüpoglu[1], Erik A. Williams[5,6], Daniel P. Cahill[7,8], Tareq A. Juratli[1,7,9]

1 Department of Neurosurgery, Faculty of Medicine and University Hospital Carl Gustav Carus, TUD Dresden University of Technology, Fetscherstraße 74, 01307 Dresden, Germany
2 Else Kröner Fresenius Center for Digital Health, Faculty of Medicine, TUD Dresden University of Technology, Dresden, Germany
3 PathoNext GmbH, Molecular Pathology, Leipzig, Germany
4 Department of Pathology, Faculty of Medicine and University Hospital Carl Gustav Carus, TUD Dresden University of Technology, Fetscherstraße 74, 01307 Dresden, Germany
5 Foundation Medicine Inc, Cambridge, MA, USA
6 Department of Pathology and Laboratory Medicine, University of Miami, Sylvester Comprehensive Cancer Center, Miami, FL, USA
7 Laboratory of Translational Neuro-Oncology, Department of Neurosurgery, Massachusetts General Hospital, Harvard Medical School, Boston, MA, USA
8 Department of Neurosurgery, Massachusetts General Hospital, Harvard Medical School, Boston, MA, USA
9 National Center for Tumor Diseases (NCT), NCT/UCC Dresden, a partnership between DKFZ, Faculty of Medicine and University Hospital Carl Gustav Carus, TUD Dresden University of Technology, and Helmholtz-Zentrum Dresden-Rossendorf (HZDR), Dresden, Germany

Corresponding author:
Tareq A. Juratli, MD
Department of Neurosurgery, University Hospital Carl Gustav Carus, TU Dresden, Germany
Email: Tareq.Juratli@ukdd.de

Supplementary Fig. S1: Methylation classifier results of four patients

Patient DD2

Generation of the copy number profile of the sample is based on conumee2 (Daenekas et. al 2024). The profile depicts log2 transformed copy number ratios (Y-axis) for chromosomes 1 through 22 (and X/Y if automatic prediction was successful). Potential gains or amplifications are represented by the positive Y-axis, while losses are depicted by the negative Y-axis. 29 CNS tumor relevant gene regions are highlighted.

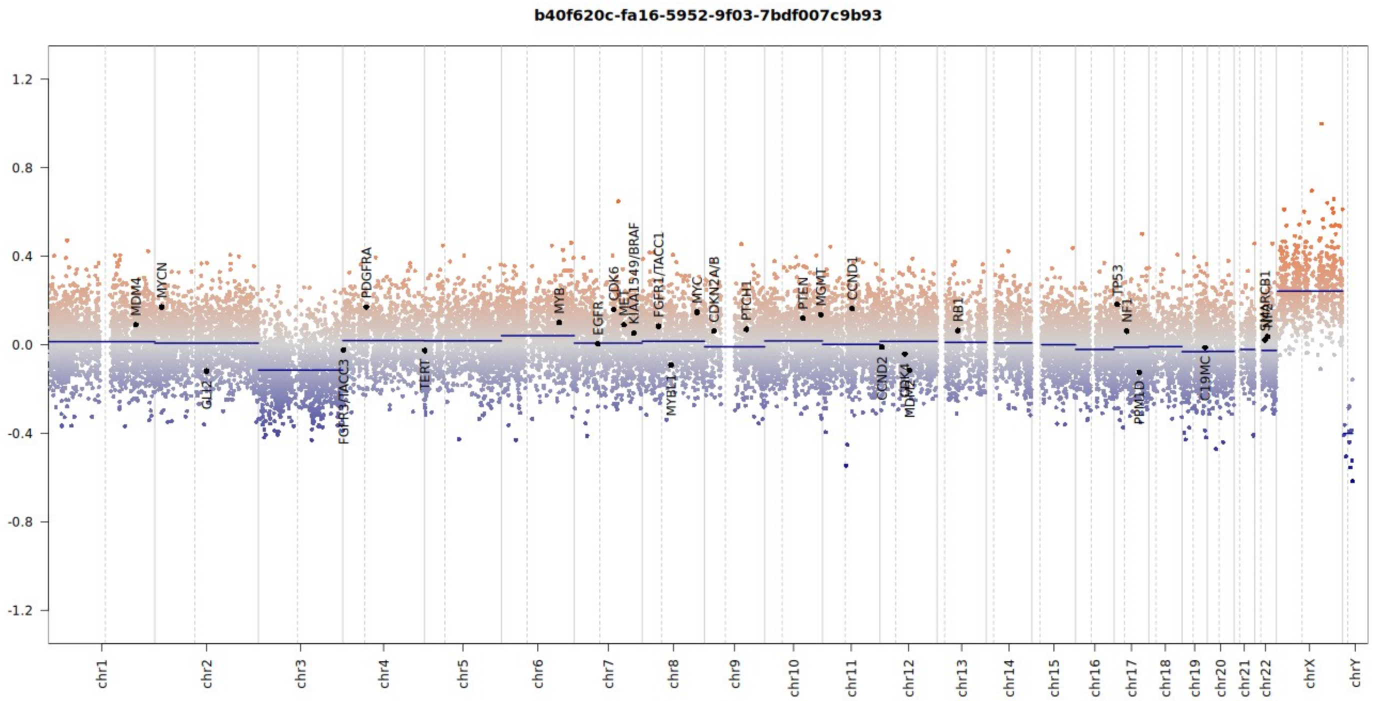

Methylation classification

Predictions				Calibrated score	Interpretation	
Ependymal tumours				0.33576	no match	✗
	supratentorial ependymoma, ZFTA fusion-positive			0.21381	no match	✗
		supratentorial ependymoma, ZFTA:RELA fusion-positive		0.19637	no match	✗
			MC Supratentorial ependymoma, ZFTA fusion-positive, subtype ZFTA-RELA fused, subclass A (novel)	0.19547	no match	✗

Legend*: ✓ Match (score >= 0.9) ✗ No match (score < 0.9): possibly still relevant for cases with low tumor content and poor DNA quality.

Hierarchy levels:

Superfamily			
	Family		
		Class	
			Subclass

*Only predictions with scores >= 0.9 are considered “classified”. Scores < 0.9 could still be relevant for cases with low tumor content or poor DNA quality. Please exercise caution in interpreting such predictions.
Only superfamily predictions with scores >=0.3 are shown, lower hierarchical levels are displayed if they are predicted with scores >=0.1

Description:

MC Supratentorial ependymoma, ZFTA fusion-positive, subtype ZFTA-RELA fused, subclass A (novel): The "mf Supratentorial ependymoma, ZFTA fusion-positive" includes "mc Supratentorial ependymoma, ZFTA fusion-positive, subtype ZFTA-RELA fused, subclass A", "mc Supratentorial ependymoma, ZFTA fusion-positive, subtype ZFTA-RELA fused, subclass B", "mc Supratentorial ependymoma, ZFTA fusion-positive, subclass C", "mc Supratentorial ependymoma, ZFTA fusion-positive, subclass D" and "mc Supratentorial ependymoma, ZFTA fusion-positive, subclass E", and represents the most common group of supratentorial pediatric ependymomas. Within this mf, subclasses A and B typically show the canonical ZFTA:RELA fusion.
Evidence Level: None

Patient DD4

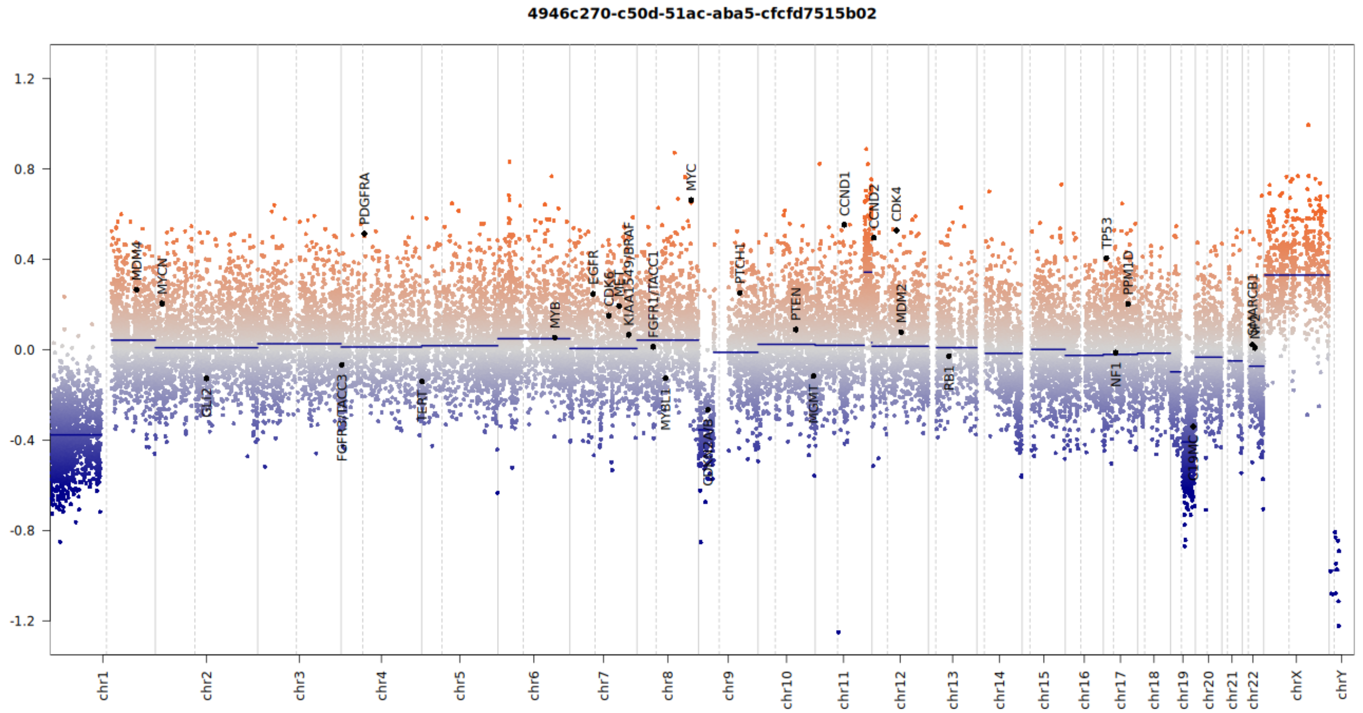

Methylation classification

Predictions				Calibrated score	Interpretation	
Adult-type diffuse gliomas				0.99518	match	✔
	diffuse glioma, IDH mutant			0.99483	match	✔
		diffuse glioma, IDH-mutant and 1p19q co-deleted [oligodendroglial type]		0.99165	match	✔
			MC Oligodendroglioma, IDH-mutant and 1p/19q-codeleted	0.99134	match	✔

Legend*: ✔ Match (score >= 0.9)

✘ No match (score < 0.9): possibly still relevant for cases with low tumor content and poor DNA quality.

Hierarchy levels:

Superfamily			
	Family		
		Class	
			Subclass

*Only predictions with scores >= 0.9 are considered “classified”. Scores < 0.9 could still be relevant for cases with low tumor content or poor DNA quality. Please exercise caution in interpreting such predictions.
Only superfamily predictions with scores >=0.3 are shown, lower hierarchical levels are displayed if they are predicted with scores >=0.1

Description:

MC Oligodendroglioma, IDH-mutant and 1p/19q-codeleted: The "msc IDH glioma, subclass 1p/19q codeleted oligodendroglioma" exclusively comprises tumours with the diagnosis oligodendroglioma, IDH-mutant and 1p/19q-codeleted grade 2 and 3. Molecularly, this class shares an IDH mutation-associated glioma CIMP, complete 1p/19q codeletion and mostly TERT promoter mutation. A missing complete 1p/19q codeletion is not compatible with this diagnosis. Cases with elevated scores for this class but no complete 1p/19q codeletion likely represent "IDH glioma, subclass astrocytoma" or "IDH glioma, subclass high grade astrocytoma ". Copy number analysis shows complete chromosome 1p and 19q loss in all cases.
Evidence Level: None

Patient DD6

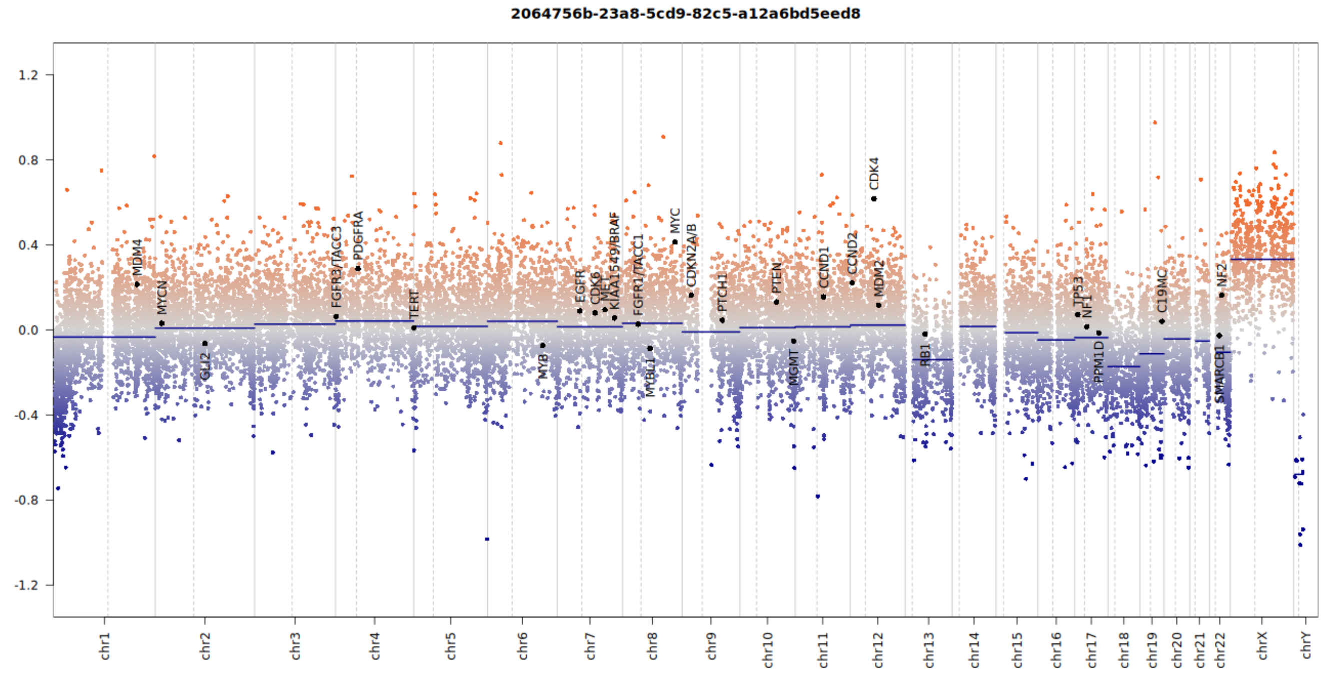

Methylation classification

Predictions				Calibrated score	Interpretation	
Ependymal tumours				0.45258	no match	✗
	supratentorial ependymoma, ZFTA fusion-positive			0.35056	no match	✗
		supratentorial ependymoma, ZFTA:RELA fusion-positive		0.33587	no match	✗
			MC Supratentorial ependymoma, ZFTA fusion-positive, subtype ZFTA-RELA fused, subclass A (novel)	0.33513	no match	✗

Legend*: ✓ Match (score >= 0.9) ✗ No match (score < 0.9): possibly still relevant for cases with low tumor content and poor DNA quality.

Hierarchy levels:

Superfamily			
	Family		
		Class	
			Subclass

*Only predictions with scores >= 0.9 are considered “classified”. Scores < 0.9 could still be relevant for cases with low tumor content or poor DNA quality. Please exercise caution in interpreting such predictions.
Only superfamily predictions with scores >=0.3 are shown, lower hierarchical levels are displayed if they are predicted with scores >=0.1

Description:

MC Supratentorial ependymoma, ZFTA fusion-positive, subtype ZFTA-RELA fused, subclass A (novel): The "mf Supratentorial ependymoma, ZFTA fusion-positive" includes "mc Supratentorial ependymoma, ZFTA fusion-positive, subtype ZFTA-RELA fused, subclass A", "mc Supratentorial ependymoma, ZFTA fusion-positive, subtype ZFTA-RELA fused, subclass B", "mc Supratentorial ependymoma, ZFTA fusion-positive, subclass C", "mc Supratentorial ependymoma, ZFTA fusion-positive, subclass D" and "mc Supratentorial ependymoma, ZFTA fusion-positive, subclass E", and represents the most common group of supratentorial pediatric ependymomas. Within this mf, subclasses A and B typically show the canonical ZFTA:RELA fusion.
Evidence Level: None

Patient DD8

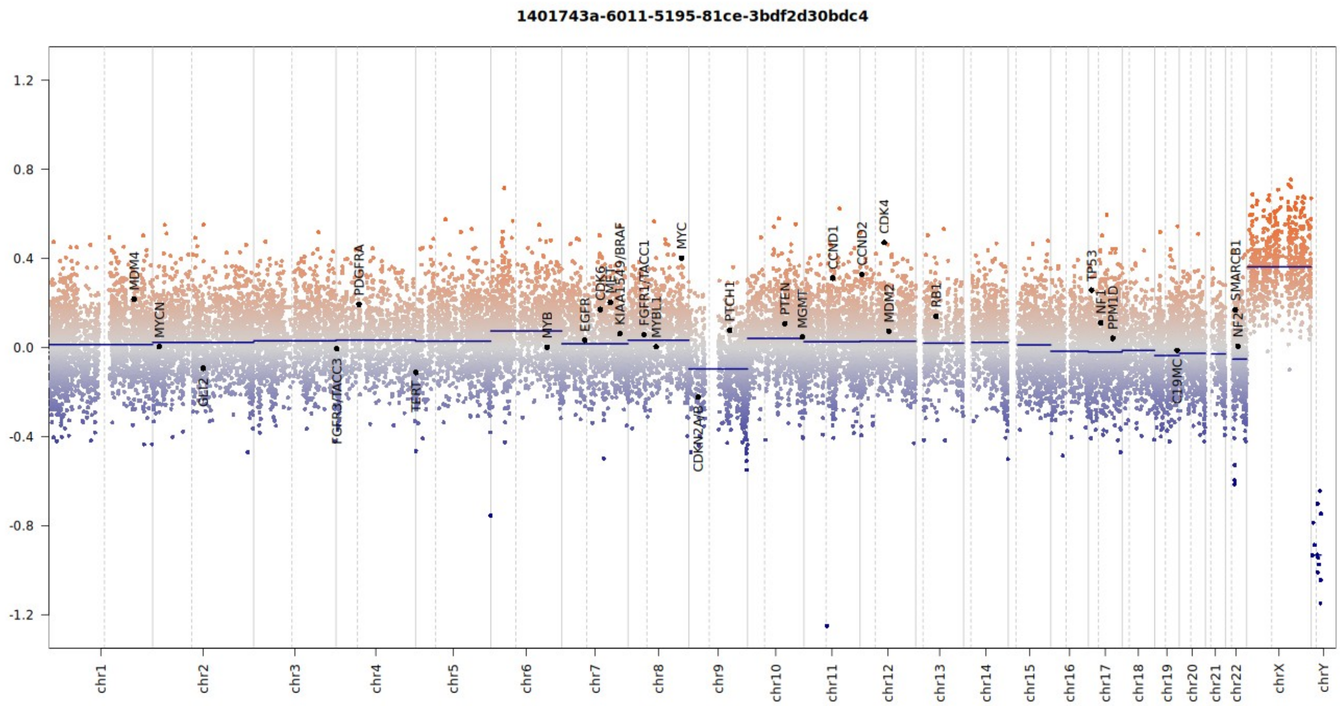

Methylation classification

No score >= 0.3
