# Supplementary material for: Comparison between molecular and histological IDH-wild-type glioblastoma and extensive subgroup analysis of IDH-wild-type astrocytic tumors without genomic glioblastoma-defining alterations
Source: J Neurooncol. 2026 Jun 10;178(2):58. doi: 10.1007/s11060-026-05637-w (PMC13253596; doi:10.1007/s11060-026-05637-w)
Supplement: Supplementary file 4 — Supplementary Material 4 [file 11060_2026_5637_MOESM4_ESM.pdf]

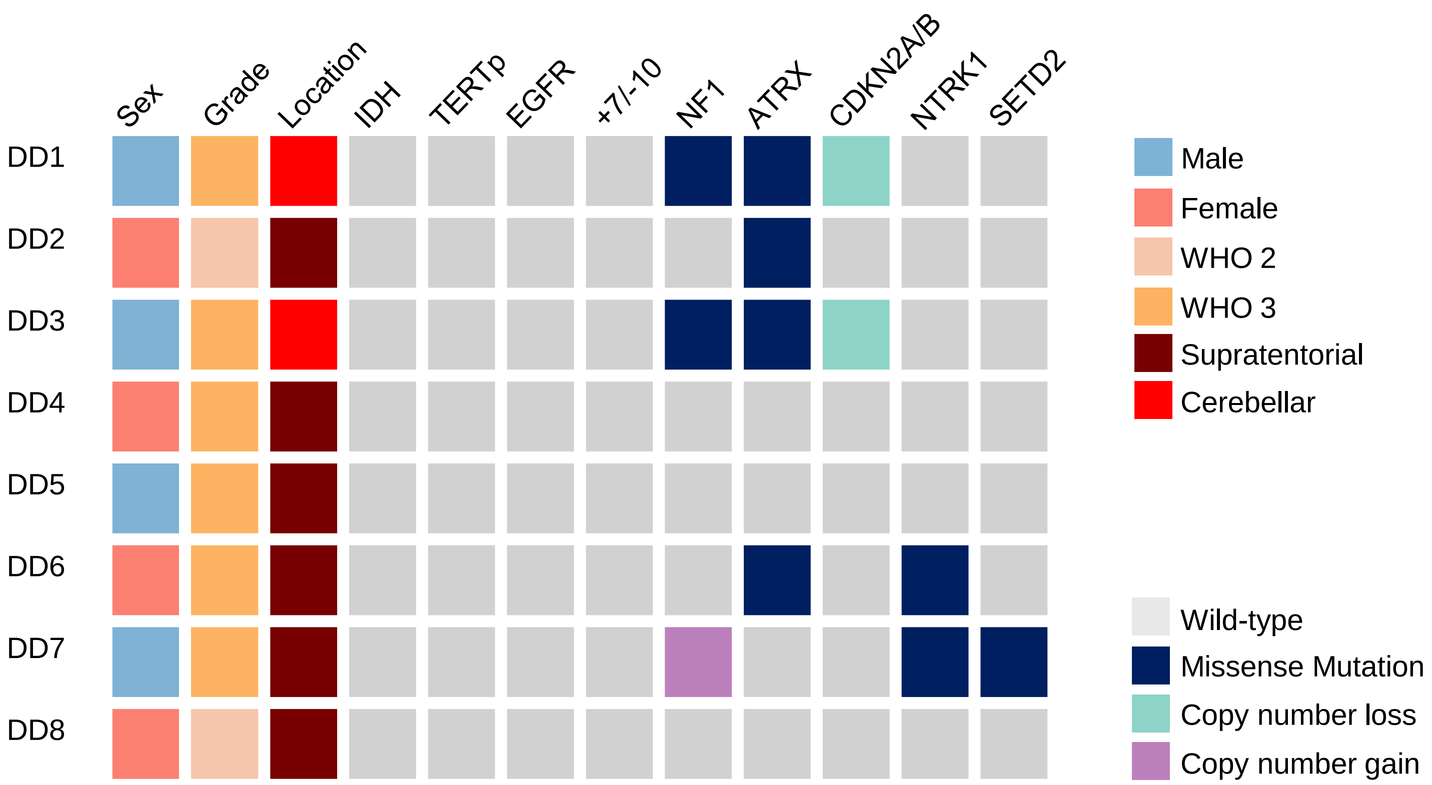

Supplementary Figure: Summary of clinical features and molecular alterations in IDH-WT, TERTp-WT tumors
